# Minocycline Nanocrystals: A New Approach for Treating Acne with Reduced Systemic Side Effects

**DOI:** 10.3390/pharmaceutics17060727

**Published:** 2025-05-31

**Authors:** Suha M. Abudoleh, Juhaina M. Abu Ershaid, Dima Lafi, Nisreen A. Dahshan, Ahmad Talhouni, Amjad Abuirmeileh

**Affiliations:** 1 Department of Basic Pharmaceutical Sciences, Faculty of Pharmacy, Middle East University, Amman 11831, Jordan; 2 Department of Applied Pharmaceutical Sciences and Clinical Pharmacy, Faculty of Pharmacy, Isra University, Amman 11622, Jordan; ad0450@iu.edu.jo (D.L.); nisreen.dahshan@iu.edu.jo (N.A.D.); 3 Department of Anesthesia, Faculty of Allied Medicine, Isra University, Amman 11622, Jordan; ahmad.talhouni@iu.edu.jo; 4 Department of Pharmacology and Clinical Pharmacy, Faculty of Pharmacy, Middle East University, Amman 11831, Jordan; armeileh@meu.edu.jo

**Keywords:** nanocrystals, minocycline, acne vulgaris, MIC

## Abstract

**Background/Objectives:** Acne vulgaris is a chronic skin infection characterized by high sebum secretion, keratosis around hair follicles, inflammation, and imbalance in androgen levels. Acne vulgaris causes permanent scars or skin pigmentation in cases of improper treatment. Oral or topical isotretinoin, contraceptives, and antibiotics are used to treat acne. Minocycline is one of the widely used tetracyclines for this purpose; it inhibits the synthesis of proteins in bacterial ribosomes. Commonly, minocycline is prescribed daily for several months for acne vulgaris. Systemic minocycline is highly distributed into body fluids, and it is associated with several side effects and antibiotic resistance. Additionally, minocycline is highly metabolized in the liver, leading to reduced bioavailability upon systemic delivery. This study aims to develop and characterize minocycline nanocrystals for targeted skin delivery and evaluate their antimicrobial efficacy in treating acne vulgaris. **Methods**: Minocycline nanocrystals were synthesized using milling or solvent evaporation techniques. Nanocrystals were characterized in terms of particle size, particle distribution index (PDI), zeta potential, and morphology. The antibacterial efficacy against *Propionibacterium acne*, *Staphylococcus aureus*, and *Staphylococcus epidermidis* was evaluated using a minimum inhibitory concentration assay (MIC) and agar well diffusion test in comparison to coarse minocycline. **Results**: Minocycline nanocrystals had a particle size of 147.4 ± 7.8 nm and 0.27 ± 0.017 of PDI. The nanocrystals exhibited a loading efficiency of 86.19 ± 16.7%. Antimicrobial testing showed no significant difference in activity between minocycline and its nanoparticle formulation. In terms of skin deposition, the nanocrystals were able to deliver minocycline topically to rat skin significantly more than free minocycline. The nanocrystal solution deposited 554.56 ± 24.13 μg of minocycline into rat skin, whereas free minocycline solution deposited 373.99 ± 23.32 μg. **Conclusions**: Minocycline nanocrystals represent a promising strategy for targeted skin delivery in the treatment of acne vulgaris, potentially reducing systemic side effects and antibiotic resistance and improving patient outcomes.

## 1. Introduction

Acne vulgaris is a common dermatological condition that is estimated to affect millions of individuals around the world [1]. This chronic condition is characterized by the formation of pustules and sometimes cysts [2]. Acne is most commonly seen in adolescents and young adults but can persist into adulthood. The pathogenesis of acne is multifactorial, including increased sebum production, hyperkeratinization of the hair follicles, inflammation, and the presence of *Cutibacterium acnes* (*C. acnes*), a key microbial agent involved in acne pathogenesis [3]. The treatment protocols for acne include topical agents such as retinoids and antibiotics, as well as oral therapies including antibiotics, hormonal treatments, and in severe cases, isotretinoin [4]. However, the use of oral antibiotics like minocycline is often associated with side effects including gastrointestinal disturbances, photosensitivity, and, notably, the development of antibiotic resistance [5].

A tetracycline derivative antibiotic, minocycline, is frequently prescribed for acne vulgaris due to its broad-spectrum antibacterial properties and ability to inhibit *C. acnes* and other skin pathogens [6]. Despite its effectiveness, the prolonged systemic use of minocycline is linked with concerns regarding bioavailability and systemic adverse effects due to its high metabolism in the liver and subsequent distribution into various body fluids [7]. These limitations highlight the need for innovative drug delivery systems that can reduce systemic exposure while maintaining efficacy at the target site—the skin.

The skin plays a vital role in protecting our body from the external environment by forming a physical and chemical barrier against exogenous materials. At the same time, it is considered an attractive drug delivery route where drugs can be applied and delivered locally or systemically [8]. However, there is a group of criteria that drugs must meet in order to be effectively delivered through the skin. Drugs must be able to penetrate the *stratum corneum* (SC), which is the most superficial layer of the skin and the layer mainly responsible for physical defense [9]. Hydrophilic drugs are unable to effectively penetrate this layer due to the hydrophobic and keratinized nature of this skin layer. In this case, hydrophilic drugs will be applied externally and stay at the outer layers of the skin without reaching the viable epidermis [10]. Minocycline hydrochloride is a hydrophilic antibiotic with a molecular weight of 493.94 g/mol [11]. This fact might challenge the effective delivery of minocycline through skin layers.

Nanotechnology, particularly the use of drug nanocrystals, has emerged as a promising approach to overcome drug delivery challenges [12]. Nanocrystal technology includes reducing the size to the nanometer scale to enhance the bioavailability and stability of poorly soluble drugs and enable more targeted delivery, thus reducing the likelihood of systemic side effects [13]. This technology has not been tested for hydrophilic molecules. In this work, nanocrystals of a hydrophilic drug (minocycline) have been prepared to evaluate the nanocrystallization effect on the antibacterial potency and delivery efficacy of hydrophilic drugs.

In this context, the development of minocycline nanocrystals for topical application offers a novel strategy to deliver the drug directly to the skin, minimizing systemic absorption and improving its therapeutic efficacy against acne vulgaris. The present study aims to prepare minocycline as a nanocrystal, testing its antibacterial ability and aiming to design targeted skin therapy to enhance drug availability with reduced systemic side effects.

## 2. Materials and Methods

### 2.1. Materials

Minocycline was purchased from (Apollo Scientific, Manchester, UK). Lutrol^®^ F127 from (BASF, Ludwigshafen, Germany), dimethyl sulfoxide (DMSO) and phosphate buffer solution (PBS) were obtained from (Sigma Aldrich, St. Louis, MO, United States). Nutrient broth and agar from (Oxoid^TM^, Basingstoke, UK). Brain heart infusion broth and agar (Biolab, Hungary) were used. *Staphylococcus aureus* (ATCC 9144), *Staphylococcus epidermidis* (ATCC 51625), and *Cutibacterium acnes* (ATCC 11827) were used. Rat skin was obtained from Isra University animal house.

### 2.2. Minocycline Nanocrystals Preparation

Minocycline nanocrystals were prepared using the antisolvent nanoprecipitation method [8]. Minocycline powder (50 mg) was dissolved in 5 mL of DMSO and placed in a sonicator until full dissolution. In a glass beaker, 100 mL of 0.5% (*w*/*v*) Pluronic^®^ F127 (PF^®^127) in distilled water (DW) was prepared. Pluronic^®^ F127 was added in this minimum concentration to allow the high loading percentage of minocycline in the formulation. Minocycline solution was added to the aqueous solution using a syringe under magnetic stirring of 300 rounds per minute (rpm). To obtain minocycline nanocrystals, the prepared nanosuspension was dried in the incubator at 37 °C for 3 days.

### 2.3. Minocycline Nanocrystals Characterization 

Firstly, minocycline nanocrystals were evaluated according to their size using a Zetasizer Nano ZS-90 instrument (Malvern^®^ Instruments, Malvern, UK). A certain amount of dried minocycline nanocrystals was added to distilled water followed by proper dilution (1:99, *v*/*v*). Nanocrystal sizes were measured in triplicate using a 90° scattering angle at 25 °C. The polydispersity index (PDI) of the nanocrystals was also measured using a Zetasizer Nano ZS-90 instrument and the same preparation steps. This measurement was performed to evaluate the distribution of the nanocrystal’s sizes. Finally, the zeta potential of the nanocrystals was measured using the Zetasizer. The same sample used for the size measurement was transferred to a folded capillary zeta cell.

### 2.4. Loading Efficiency

Minocycline weight per nanocrystal weight was obtained to evaluate the loading efficiency of the nanocrystals. About 50 mg of minocycline nanocrystals were dissolved in DMSO. Further, samples were vortexed and then centrifuged at 10,000× *g* for 10 min. Finally, the supernatant was diluted with DMSO and absorbance was measured using an ultraviolet (UV) spectrophotometer at 346 nm wavelength. A calibration curve was conducted over a concentration range of 3.125–50 µg/mL. Loading efficiency was measured according to Equation (1). The calibration method was used to quantify minocycline in the formulation; the R-value was 0.9999, and the data is presented as the mean ± SD.

(1) $$Loading\;efficiency\%=\frac{Minocycline\;amount}{Total\;amount\;of\;the\;formulation}\times \;100\%$$

### 2.5. Antibacterial Activity

#### 2.5.1. Antibiotic Solubility

Minocycline and minocycline nanocrystals were dissolved in distilled water and then filtered using a 0.45 µm syringe filter. Equal concentrations of minocycline were used for free drug solution and NCs solution.

#### 2.5.2. Agar Well Diffusion Method

The antimicrobial activities of minocycline and minocycline nanocrystals were tested using the agar well diffusion method [14]. The test was performed against *S. aureus* (ATCC 9144), *S. epidermidis* (ATCC 51625), and *C. acnes* (ATCC 11827). Bacterial concentrations were adjusted to 0.5 McFarland, and 100 µL of each bacterial growth was evenly spread on the surface nutrient agar for *S. aureus* and *S. epidermidis* and brain heart infusion agar (BHI) for *C. acnes* [15,16].

Sterile cork borers with an 8 mm diameter were used to create agar wells. Each well was filled with 200 µL of either minocycline (33.33 mg/mL) or minocycline nanocrystal (33.33 mg/mL). The plates were then incubated at 37 °C for 18 h, under aerobic conditions for *Staphylococci* and strict anaerobic conditions for *C. acnes*. The diameter of inhibition zones surrounding each well was measured [17]. The experiment was conducted in triplicate.

#### 2.5.3. Minimum Inhibitory Concentration (MIC)

The MIC was determined following the method described by [15,18] using a 96-well plate assay. Briefly, 100 µL of nutrient broth was added to each well for *S. aureus* and *S. epidermidis*, while brain heart infusion (BHI) broth was used for *C. acnes*.

Then, 100 µL of minocycline and minocycline nanocrystals were separately added to the first well and serially diluted to the following concentrations: 1042 µg/mL, 520.83 µg/mL, 260.41 µg/mL, 130.21 µg/mL, 65.1 µg/mL, 32.55 µg/mL, 16.28 µg/mL, 8.14 µg/mL, 4.07 µg/mL, 2.03 µg/mL, 1.02 µg/mL, and 0.51 µg/mL. Then, 100 µL of bacterial suspension (1 × 10^6^ CFU/mL) was added to each well.

The plates were incubated at 37 °C for 18 h. The MIC was recorded as the lowest concentration at which no visible bacterial growth was observed.

### 2.6. Ex Vivo Skin Deposition and Permeation Study

To investigate the ability of NCs to deliver minocycline topically to the skin, an ex vivo skin deposition study was performed using full-thickness rat skin obtained from the Isra University animal house. Rat skin was fully cleaned, shaved, and stored at −70° C until usage. This experiment was conducted using Franz diffusion cells. Each receiver compartment was filled with 16 mL of PBS (pH = 7.4) and a magnetic stirrer to ensure continuous circulation in the system. Skin pieces were attached to the donor compartment by glue to ensure fixation during the study. Using a micropipette, 500 µL each of NC solution and minocycline solution (same concentrations) was placed on top of Franz cells to allow skin deposition. The entire system was covered up with Parafilm^®^.M (Amcor, Zürich, Switzerland) to prevent vaporization. The temperature was kept at 37 ± 1 °C. At predetermined time points (0.5, 1, 3, 6, 24 h) 0.5 mL of the reservoir compartment was withdrawn through the arm outlet of the Franz cell while replacing it immediately with the same volume of fresh release media. Samples were diluted, vortexed, and analyzed using a UV spectrophotometer. For skin samples, after 24 h of application, the skin was cut into small pieces and 3 mL of a mixture of DMSO–distilled water (1:2) was added. Samples were sonicated for 30 min, vortexed, and centrifuged at 4500× *g* rpm for 10 min. Supernatants were further diluted and analyzed using a UV spectrophotometer.

### 2.7. Statistical Analysis

Statistical analysis was performed using GraphPad Prism software (version 10.0; GraphPad Software, San Diego, CA, USA). The data were processed using Microsof^t®^ Excel^®^ 2016. Independent sample *t*-tests were used for comparing groups, and the significance level was set at *p* < 0.05.

## 3. Results and Discussion

### 3.1. Minocycline Nanocrystals Characterization

**Figure 1:**
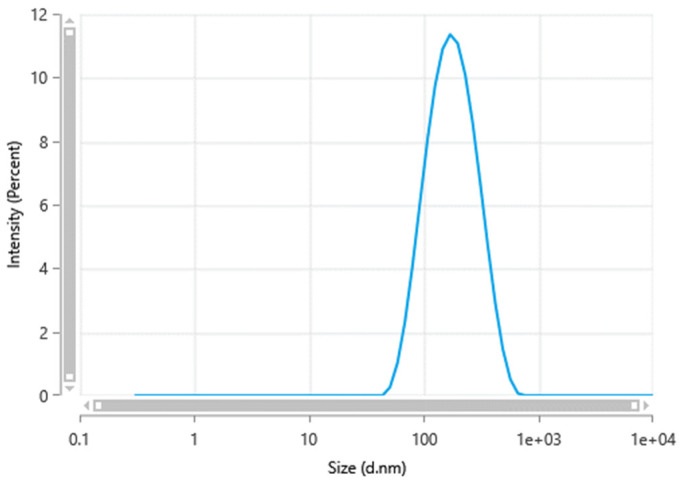
Minocycline nanocrystal size measured by a Zetasizer Nano ZS-90 instrument (Malvern^®^ Instruments, Malvern, UK).

Minocycline nanocrystals were prepared using the antisolvent nanoprecipitation method. This method offers a simple, direct, and easy approach to preparing nanocrystals [19,20]. In simple terms, this method includes dissolving the drug in a suitable solvent to prepare a solution, then adding this solution to nonsolvent or antisolvent to allow drug displacement to the antisolvent under continuous stirring, enabling the formation of finely dispersed nanocrystals [21,22]. As previously reported, this method has been applied to prepare nanocrystals of various antibiotics [19,23]. Minocycline nanocrystals showed a particle size of 147.4 ± 7.8 nm, as shown in Figure 1. Nanocrystals are defined as a type of nanoparticle with particle size between 1 and 1000 nm [22,24]. Despite the fact that there is debate about the classification of nanocrystals according to size, in pharmaceutics, nanocrystals are defined as having a particle size of less than 1000 nm [20,25]. However, in colloid chemistry, particles should have a size below 100 nm to be classified as nanoparticles [21,26]. Refining the size in the nanometer range increases the surface area, which affects various parameters such as dissolution [19,27]. The nano size facilitates the transdermal delivery of molecules by enhancing the penetration through skin layers [22,28].

Further, nanocrystals were characterized based on particle size distribution by measuring the PDI. PDI was used as an indicator of the physical stability of the nanocrystal suspension. In this work, minocycline nanocrystals had a PDI of 0.27 ± 0.017. Generally, a PDI of more than 0.5 is considered an indication of a wide size distribution [25,29]. Having a narrow size distribution is an effective way to support the physical stability of nanocrystals. A wide size distribution increases the risk of Ostwald ripening, which decreases stability by reducing the solubility, dissolution rate, and bioavailability [26,30].

**Figure 2:**
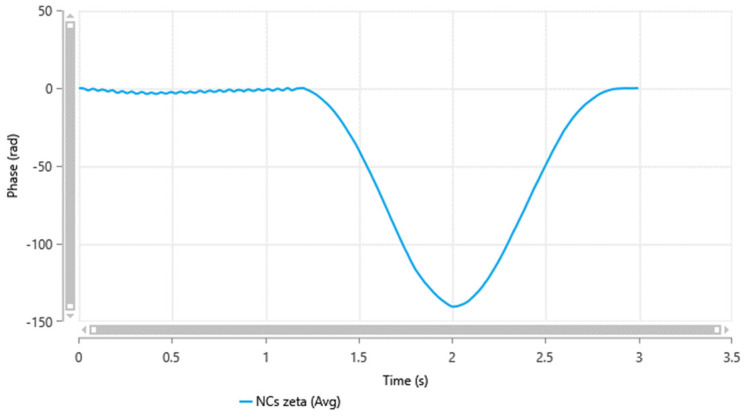
Minocycline nanocrystals zeta potential measured by a Zetasizer Nano ZS-90 instrument (Malvern^®^ Instruments, Malvern, UK).

Zeta potential measures the outer charge of the particles’ shear surfaces [27,31]. It is performed to evaluate the physical stability of the nanocrystals. Nanocrystals with low absolute zeta potential values are more susceptible to aggregation and losing their physical stability [28,32]. The zeta potential of the minocycline nanocrystals, as shown in Figure 2, was −12.29 ± 0.6 mV, which is similar to a previously reported study where PF^®^127 was used to stabilize nanocarriers [33,34]. It is worth mentioning that the PF^®^127 percentage in the formulation plays a role in particle surface charges [29,33]. The absolute value of zeta potential increases with increasing concentrations of surfactant or polymers in the formulation [30,34].

### 3.2. Loading Efficiency

Minocycline content in the nanocrystals was determined to evaluate the loading efficiency percentage. Nanocrystals showed a loading efficiency of 86.19 ± 16.7% *w*/*w*. One of the major advantages of nanocrystals over other nanosystems is that they are mostly prepared from pure drugs with low surfactant concentrations [19,23]. This advantage might enhance the antibacterial efficacy of NCs due to the increased localization of minocycline [31,35].

### 3.3. Agar Well Diffusion Method

**Figure 3:**
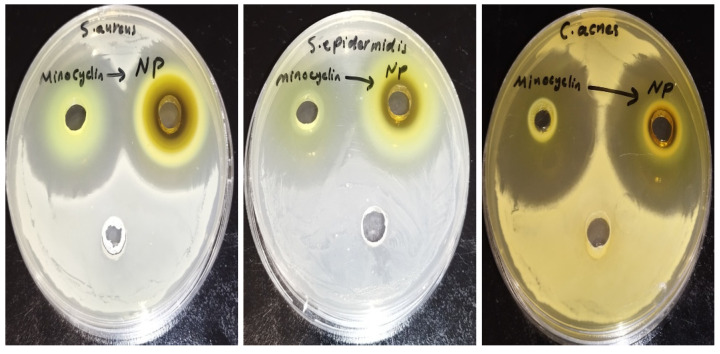
The inhibition zone of minocycline and minocycline nanocrystals against *S. aureus*, *S. epidermidis*, and *C. acne*.

The agar well diffusion method revealed no significant difference in activity between the minocycline and the NC formulation on *C. acnes* (*p*-value: 0.578), *S. epidermis* (*p*-value: 0.071), and *S. aureus* (*p*-value: 0.179) according to an unpaired *t*-test, as presented in Table 1 and illustrated in Figure 3.

### 3.4. MIC

The MIC results are shown in Table 2. No changes in MIC values were observed against the tested bacteria for free minocycline and NCs. The MIC values were difficult to determine exactly (<0.51 µg/mL). The results of MIC showed that the preparation method of minocycline NCs did not affect the activity of minocycline against the three studied bacteria.

### 3.5. Ex Vivo Skin Deposition and Permeation Study

**Figure 4:**
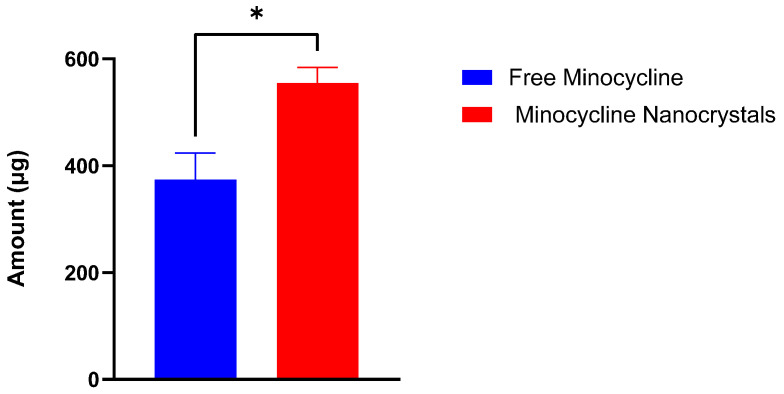
Minocycline amount (µg) deposited in rat skin after 24 h of the application of free minocycline solution and minocycline nanocrystals in a Franz diffusion study. (mean ± SD, *n* = 3). *: significantly different than each other (*p*-value = 0.0103).

**Figure 5:**
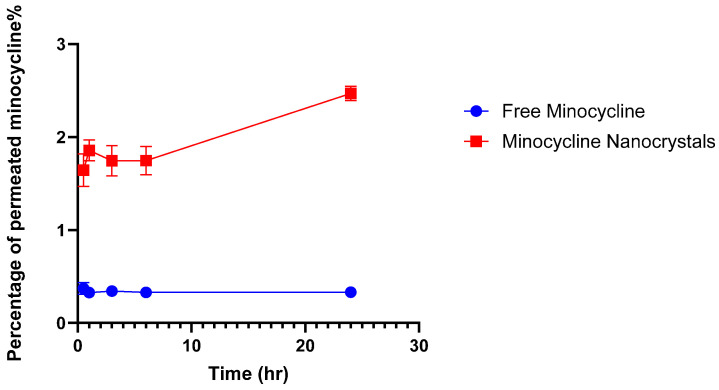
Percentage of permeated minocycline through rat skin into the reservoir compartment in Franz diffusion cells. (mean ± SD, *n* = 3).

The targeted delivery of minocycline into the skin includes depositing the drug locally while ensuring minimum drug permeation into the systemic circulation [36]. This experiment was performed to evaluate the potential of an NC formulation to provide the targeted delivery of minocycline. NCs and free minocycline solutions were each placed on the top of the outer surface of the skin and maintained for 24 h in the Franz cells setup. After completing the experiments, skin pieces from both groups were processed and analyzed to quantify minocycline using a UV spectrophotometer. NCs were able to deposit 554.56 ± 24.13 μg whereas free minocycline deposited 373.99 ± 23.32 μg. The difference between the two values was significant (*p*-value = 0.0103), as shown in Figure 4, indicating the higher ability of NCs to deliver minocycline locally. In terms of drug permeability, minocycline permeation through skin layers into the reservoir compartment was evaluated from the free drug solution and nanocrystal solution using a UV spectrophotometer as shown in Figure 5. Minocycline permeability through rat skin layers after 24 h of application, calculated as a percentage of the total minocycline placed on top of the skin, was 0.33 ± 0.001% for free drug and 2.46 ± 0.076% for NCs. Both solutions showed a minimum drug permeability which can be justified due to the hydrophilic nature of minocycline. This topical application of minocycline deposited, as a percentage of the total applied drug, was about 11.09 ± 0.48% for NCs and 7.47 ± 0.81% for free minocycline with minimum drug permeation. NCs showed a superior drug deposition compared with free minocycline and minocycline foam, as was previously reported [37]. This might be due to the small size of the NCs, which facilitated the drug’s penetration and distribution through skin layers.

### 3.6. Current Findings and Future Prospects

This research focused on developing nanocrystals of minocycline to provide an effective approach to delivering antibiotics locally to the skin. Minocycline nanocrystals had a similar antimicrobial potency against tested bacteria compared to free minocycline while NCs showed superior drug deposition into the skin. However, this concept needs further investigations to evaluate the physical properties of the nanocrystals and to address any potential chemical interaction between minocycline and the excipients used. Further studies such as in vivo and toxicity studies are also required.

## 4. Conclusions

In conclusion, the preparation of minocycline NCs offers a novel and efficient approach to topical acne treatment, with special emphasis on the fact that nanocrystal preparation and characterization do not affect the efficacy of the minocycline against the tested bacteria in this study. In contrast, the NC preparation enhanced minocycline deposition within skin layers, which supports its application for targeted drug delivery. This study provides a foundation for future research on nanotechnology-based acne treatments, emphasizing the need for further experiments for validation while looking for safer, effective treatment. Further investigation such as in vivo, stability, and toxicity studies should be conducted to investigate the efficacy and safety of this formulation in vivo *and* on cells.

## Figures and Tables

**Table 1:** The inhibition zone of minocycline and minocycline nanocrystals against *S. aureus*, *S. epidermidis*, and *C. acnes*. The diameter in cm ± SD, *n* = 3.

Sample	*S. aureus* (ATCC 9144)	*S. epidermidis* (ATCC 51625)	*C. acnes* (ATCC 11827)
Minocycline 33.33 mg/mL	4.333 ± 0.125	4.1 ± 0.082	4.4 ± 0.082
Minocycline nanocrystal 33.33 mg/mL	4.167 ± 0.125	3.967 ± 0.047	4.433 ± 0.047

**Table 2:** The MIC values of minocycline and minocycline nanocrystals against the tested bacteria.

Sample	*S. aureus* (ATCC 9144)	*S. epidermidis* (ATCC 51625)	*C. acnes* (ATCC 11827)
Minocycline	<0.51 µg/ml	<0.51 µg/ml	<0.51 µg/ml
Minocycline nanocrystal	<0.51 µg/ml	<0.51 µg/ml	<0.51 µg/ml

## Data Availability

The original contributions presented in this study are included in the article. Further inquiries can be directed to the corresponding authors.

## Abbreviations

The following abbreviations are used in this manuscript:

MIC	Minimum inhibitory concentration
PDI	Particle distribution index
BHI	Brain heart infusion broth
